# Targeting Hyperuricemia and NLRP3 Inflammasome in Gouty Arthritis: A Preclinical Evaluation of Allopurinol and Disulfiram Combination Therapy

**DOI:** 10.3390/ph18050762

**Published:** 2025-05-21

**Authors:** Yahya I. Asiri, Manimekalai Pichaivel, Selva Prasanthi Parameshwaran, Krishnaraju Venkatesan, Saud Alqahtani, Taha Alqahtani, Rehab Ahmed, Hassabelrasoul Elfadil, Mahmoud Elodemi, Shaimaa Genena, Durgaramani Sivadasan, Premalatha Paulsamy

**Affiliations:** 1 Department of Pharmacology, College of Pharmacy, King Khalid University, Abha 62521, Saudi Arabia; yialmuawad@kku.edu.sa (Y.I.A.); saalqhtany@kku.edu.sa (S.A.); ttaha@kku.edu.sa (T.A.); 2 Department of Pharmacology, Swamy Vivekanadha College of Pharmacy, Elayampalayam, Namakkal 637205, Tamil Nadu, India; mekalaai@svcop.ac.in (M.P.); prasanthiparameshwaran1998@gmail.com (S.P.P.); 3 Division of Microbiology, Immunology and Biotechnology, Department of Natural Products and Alternative Medicine, Faculty of Pharmacy, University of Tabuk, Tabuk 71491, Saudi Arabia; rahmed@ut.edu.sa (R.A.); habdelgadir@ut.edu.sa (H.E.); 4 Pharmacology Department, Faculty of Medicine, University of Tabuk, Tabuk 71491, Saudi Arabia; malodami@ut.edu.sa; 5 Clinical Biochemistry Department, University of Tabuk, Tabuk 71491, Saudi Arabia; sgenena@ut.edu.sa; 6 Department of Pharmaceutics, College of Pharmacy, Jazan University, Jazan 45142, Saudi Arabia; dsivadasa@jazanu.edu.sa; 7 College of Nursing, Mahalah Branch for Girls, King Khalid University, Abha 62521, Saudi Arabia; pponnuthai@kku.edu.sa

**Keywords:** allopurinol, disulfiram, inflammatory mediators, MSU crystal, potassium oxonate, uric acid

## Abstract

**Background/Objectives:** Gouty arthritis (GA) is a chronic inflammatory condition characterized by hyperuricemia and NLRP3 inflammasome activation, leading to joint damage and systemic inflammation. Although allopurinol (ALP), a xanthine oxidase inhibitor, effectively lowers serum urate levels, it has limited anti-inflammatory effects. This study investigated whether combining disulfiram (DSF), a known NLRP3 inflammasome inhibitor, with ALP enhances therapeutic outcomes in a rat model of gout. **Methods:** Thirty male Albino Wistar rats (150–200 g) were randomly assigned to five groups (*n* = 6): control, disease control, ALP-treated, DSF-treated, and ALP + DSF combination. Hyperuricemia was induced using potassium oxonate, followed by MSU crystal injection to trigger acute gout. Treatment lasted 30 days. Efficacy was assessed through clinical scoring, paw swelling, serum uric acid levels, ELISA-based cytokine profiling (IL-1β, TNF-α, IL-6), renal function tests, radiography, and histopathology. **Results:** Combination therapy with ALP + DSF significantly reduced paw swelling (*p* < 0.05), inflammation index (*p* < 0.001), serum uric acid (*p* < 0.001), and pro-inflammatory cytokines compared to monotherapy. Histopathology revealed preserved synovial architecture and reduced inflammatory infiltration. Radiographic imaging showed attenuated soft tissue swelling and joint erosion. Renal function markers were also improved in the combination group. **Conclusions:** The combination of ALP and DSF provided superior anti-inflammatory and urate-lowering effects compared to individual treatments. These findings support the potential of disulfiram as an adjunct to conventional ULTs in gout management through dual modulation of urate metabolism and inflammasome-driven inflammation.

## 1. Introduction

Gout is a chronic inflammatory arthritis that develops due to sustained hyperuricemia, which precedes and promotes the formation of monosodium urate (MSU) crystals in joints and soft tissues. Consistent elevation of serum uric acid levels surpasses its solubility threshold, leading to urate crystal deposition, which triggers gout flares through innate immune activation [1,2,3]. While hyperuricemia is the necessary biochemical precursor to gout, its etiology is multifactorial, involving increased urate production and impaired excretion [4]. Humans, lacking the enzyme uricase, convert purines to uric acid as the final catabolic product [5]. Overproduction can arise from purine-rich diets, high fructose intake, alcohol use, obesity, or rapid cell turnover. In contrast, underexcretion, more commonly implicated, is often due to dysfunction in renal and intestinal urate transporters such as URAT1, GLUT9, ABCG2, and OATs, which are influenced by genetic polymorphisms [6]. Although not all individuals with hyperuricemia develop gout, additional local factors, including reduced joint temperature, low pH, high sodium concentration, and preexisting joint damage, facilitate MSU crystal formation [6].

These crystals function as damage-associated molecular patterns (DAMPs), which, upon phagocytosis by macrophages, activate the NLRP3 inflammasome through a two-step process: priming via NF-κB signaling and activation via lysosomal rupture, potassium efflux, and mitochondrial ROS. This leads to caspase-1 activation and cleavage of pro-IL-1β into active IL-1β, initiating a robust inflammatory cascade. This response involves macrophages, neutrophils, and synovial fibroblasts, which contribute to the amplification of inflammation and joint tissue remodeling. The resulting cytokine storm promotes vasodilation, further neutrophil recruitment, and local tissue injury. If unresolved, chronic hyperuricemia may result in tophi formation and progressive joint destruction [7,8,9,10,11,12]. Beyond joints, hyperuricemia contributes to systemic effects: uric acid, while physiologically antioxidant, exhibits pro-oxidant and pro-inflammatory behavior at pathological levels, exacerbating endothelial dysfunction and promoting comorbidities such as hypertension, insulin resistance, and renal disease [4,13]. Moreover, recent studies suggest that gut dysbiosis may influence urate metabolism and immune responses [14], further positioning gout as a systemic metabolic–inflammatory disorder rather than a localized joint disease.

Conventional gout treatments focus on two primary objectives: (1) lowering serum urate levels to prevent MSU crystal formation and (2) controlling acute inflammation during flares [15]. Urate-lowering drugs (ULDs), such as allopurinol and febuxostat, reduce hyperuricemia by inhibiting xanthine oxidase, whereas NSAIDs, colchicine, and corticosteroids are used to manage inflammation during acute attacks [16]. However, while allopurinol effectively controls urate levels, it does not target the inflammatory pathways that drive acute flares and joint destruction. This has led to growing interest in inflammasome-targeted therapies, particularly NLRP3 inhibitors, which have emerged as promising alternatives for treating inflammation in gout beyond conventional NSAIDs and steroids [17].

Recent research underscores the pivotal role of the NLRP3 inflammasome in mediating gouty inflammation. Activation of NLRP3 leads to caspase-1 activation, IL-1β secretion, and pyroptosis, amplifying the inflammatory response [18]. Several studies highlight novel small-molecule NLRP3 inhibitors, such as Dapansutrile (OLT1177™), MCC950, and cucurbitacin B, which effectively reduce MSU-induced inflammation in experimental gout models [19,20]. These inhibitors block inflammasome assembly by inhibiting the oligomerization of Apoptosis-associated Speck-like protein containing a CARD (ASC), thereby preventing downstream cytokine release and reducing joint inflammation and tissue damage [21]. However, most NOD-like receptor family pyrin domain-containing 3 (NLRP3) inhibitors remain in early-stage clinical trials, and their long-term safety and efficacy in gout patients are yet to be fully established.

One promising Food and Drug Administration (FDA) approved drug with NLRP3 inhibitory properties is disulfiram (DSF). Traditionally used for alcohol-use disorder, DSF has recently been identified as a potent inhibitor of NLRP3 activation, preventing inflammasome assembly and IL-1β secretion [22]. Studies demonstrate that DSF effectively suppresses MSU-induced inflammation, reduces neutrophil recruitment, and alleviates joint swelling in animal models of gout [23]. Its ability to suppress pyroptosis and limit tissue damage makes it an attractive candidate for repurposing in inflammatory diseases [24]. Given its long-standing clinical use and established safety profile, DSF presents a viable alternative for suppressing inflammasome activation in gout without the side effects associated with NSAIDs and corticosteroids.

Rodent models remain instrumental in evaluating novel gout therapeutics. Potassium oxonate (PO), a uricase inhibitor, induces hyperuricemia in rats, mimicking human metabolic conditions. At the same time, intra-articular MSU crystal injection triggers acute gouty arthritis, providing a reliable preclinical model for testing anti-inflammatory and urate-lowering agents [25]. Using this model, we aim to investigate the therapeutic efficacy of combining ALP with DSF to achieve both urate reduction and inflammasome inhibition. Our study hypothesizes that this dual-targeted approach will provide superior disease control by addressing hyperuricemia and NLRP3-mediated inflammation, thereby reducing gout severity, preventing recurrent flares, and preserving joint integrity.

Given the rising interest in NLRP3 inhibitors and the need for effective gout therapies beyond urate-lowering agents, this study evaluates the synergistic potential of allopurinol and disulfiram in treating hyperuricemia and gouty arthritis. Targeting both urate metabolism and inflammasome-driven inflammation could provide an improved therapeutic strategy for better disease management.

## 2. Results

### 2.1. Physical Evaluation

#### 2.1.1. Effect of ALP with DSF on Body Weight

Body weight was recorded at the beginning and end of the experimental period. As shown in Table 1, rats in the gouty arthritis (GAR) control group (Group II) showed a noticeable reduction in final body weight (128.5 ± 2.232 g) compared to baseline (180.7 ± 1.430 g), indicating systemic effects of potassium oxonate-induced hyperuricemia and inflammation. Treatment with allopurinol alone (Group III) mitigated weight gain, resulting in a final weight of 159.7 ± 1.358 g. Groups receiving disulfiram (Group IV) or the combination therapy (Group V) exhibited stable body weights compared to baseline (177.3 ± 1.116 g and 179.7 ± 1.453 g, respectively). The combination of ALP + DSF (Group V) showed a statistically significant increase (*p* < 0.01) in body weight compared to the ALP-only group (Group III), indicating improved systemic recovery. No signs of weight loss or cachexia were observed in any treatment group, confirming the tolerability and therapeutic benefit of the interventions.

#### 2.1.2. Effect of ALP with DSF on Paw Swelling

**Figure 1:**
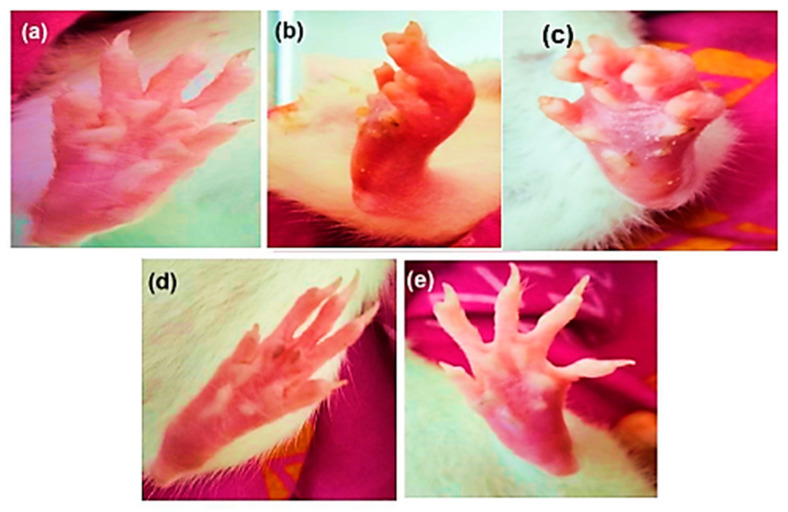
Morphological representation of the rat paw after sub-plantar administration of MSU crystals: (**a**) normal control, (**b**) disease control, (**c**) ALP (10 mg/kg), (**d**) DSF (50 mg/kg), and (**e**) ALP (10 mg/kg) + DSF (50 mg/kg).

Rats were injected with MSU crystals made using uric acid and sodium hydroxide and diluted in ordinary saline. All rats except the normal control exhibited substantial swelling, redness, and deformity at their ankle joints following the injection of MSU crystals (Table 2, Figure 1). All rats had their paw edema evaluated on days 21, 26, and 30.

On day 26, the Group V animals treated with the ALP and DSF combination exhibited a significant reduction in paw swelling (*p* < 0.05) compared to Group III. Additionally, Group V showed a significant (*p* < 0.05) decrease in paw swelling compared to Group IV. On day 30, ALP and DSF-treated Group V animals significantly reduced paw swelling (*p* < 0.05) compared to Group III. Additionally, Group V showed a notable decrease in paw swelling compared to Group IV, indicating that the combination therapy of ALP and DSF brought the paw swelling closer to normal levels.

#### 2.1.3. Effect of ALP with DSF on Inflammation Index

**Figure 2:**
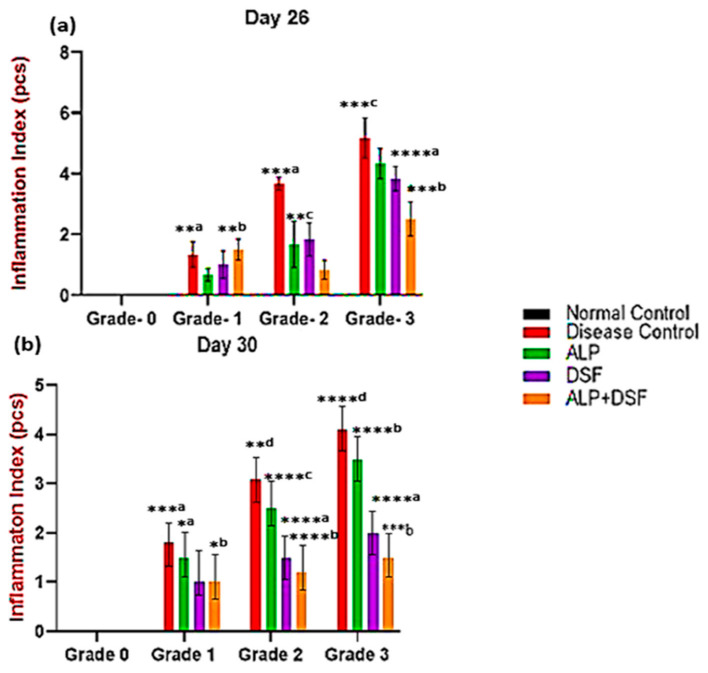
Effect of ALP with DSF on the inflammation index in PO and MSU-induced GAR on day 26 (**a**) and day 30 (**b**). Values are expressed as mean ± SEM, *n* = 6. Data were analyzed by One-way ANOVA followed by Tukey’s multiple comparison test. *^a^ *p* < 0.05—ALP vs. DSF, *^b^ *p* < 0.05—ALP vs. ALP + DSF, **^a^ *p* < 0.05—Disease Control vs. ALP + DSF, **^b^ *p* < 0.05—ALP vs. ALP + DSF, **^c^ *p* < 0.05—ALP vs. DSF, **^d^ *p* < 0.05—Disease Control vs. ALP, ***^a^ *p* < 0.001—Disease Control vs. ALP + DSF, ***^b^ *p* < 0.001—DSF vs. ALP + DSF, ***^c^ *p* < 0.001—Disease Control vs. ALP, ****^a^ *p* < 0.0001 DSF vs. ALP + DSF, ****^b^ *p* < 0.0001—ALP vs. ALP + DSF, ****^c^ *p* < 0.0001—ALP vs. DSF, ****^d^ *p* < 0.0001—Disease Control vs. ALP + DSF.

The MSU crystal injection caused the rats to exhibit many arthritis symptoms gradually. After 12 h, arthritis was present. So, the inflammation index scores were assessed on days 26 and 30 (Figure 2). On day 26, there were significant changes in inflammation index (Grade 1 and 2) in Group V compared to Group III and IV. In Grade 3, ALP with DSF-treated Group V animals revealed a significant reduction in the inflammation index (*p* < 0.05) compared to Group III.

On day 30, Group III exhibited a mild reduction in inflammation but did not achieve full resolution. Group IV showed a more pronounced decrease in inflammation, while Group V demonstrated the most effective outcome, with inflammation nearly completely resolved. In Grade 3, ALP and DSF-treated Group V animals displayed a significant reduction in the inflammation index compared to Group III (*p* < 0.001) and Group IV (*p* < 0.01). The combination therapy in Group V provided the most substantial anti-inflammatory effect, almost eliminating inflammation. While ALP treatment alone (Group III) resulted in only a mild reduction without complete resolution, and DSF treatment alone (Group IV) led to a significant decrease in inflammation, the synergistic action of ALP and DSF in Group V produced the most remarkable outcome. This highlights their combination as the most effective strategy for substantial inflammation control.

#### 2.1.4. Effect of ALP with DSF on the Dysfunction Index

The MSU crystal injection caused the rats to exhibit many arthritis symptoms gradually. The occurrence of arthritis was obvious after 12 h. Hence, the dysfunction index scores on days 26 and 30 (Table 3).

The dysfunctional index evaluates the impact of different treatments on inflammation or disease severity by categorizing responses into four grades (Grade 0 to Grade 3). The Normal Control group (Group I) exhibited no inflammation, as indicated by consistent 0.00 ± 0.00 values across all grades. In contrast, the Disease Control group (Group II) showed significantly higher values in Grade 2 and Grade 3, confirming that subjects developed inflammation when untreated. Group III exhibited moderate inflammation among the treatment groups, suggesting partial effectiveness. Groups IV and V showed a noticeable reduction in Grade 2 and Grade 3 scores, indicating improved efficacy in controlling inflammation. The most effective treatment appeared to be Group V, which demonstrated the lowest values in the higher grades, suggesting a significant reduction (*p* < 0.01) in disease severity. These results indicate that while untreated subjects developed inflammation, the tested treatments helped alleviate symptoms to varying degrees, with Group V emerging as the most effective intervention.

### 2.2. Biochemical Estimation

#### 2.2.1. Effect of ALP with DSF on Serum Uric Acid

**Figure 3:**
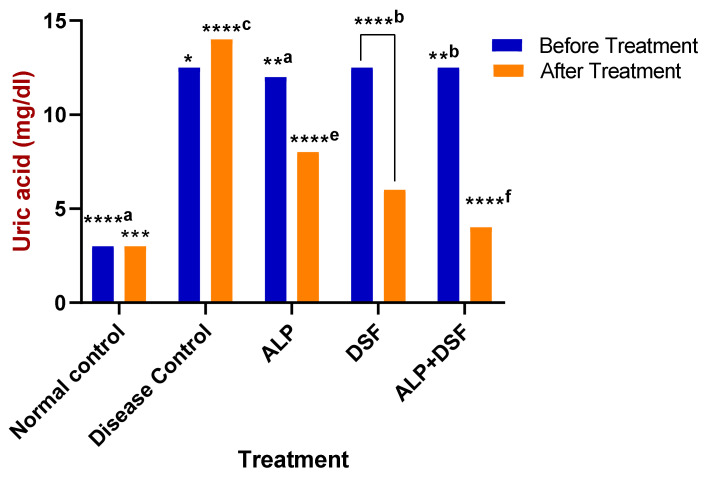
Effect of ALP with DSF on serum uric acid in PO and MSU-induced GAR before and after treatment. * *p* < 0.05—ALP vs. ALP + DSF, **^a^ *p* < 0.05 ALP vs. DSF, **^b^ *p* < 0.05 ALP vs. ALP + DSF, *** *p* < 0.001 Normal Control vs. ALP + DSF, ****^a^ *p* < 0.0001 Normal Control vs. ALP + DSF, ****^b^ *p* < 0.0001 Normal Control vs. DSF, ****^c^ *p* < 0.0001 Disease Control vs. ALP + DSF, ****^e^ *p* < 0.0001 ALP vs. DSF, ****^f^ *p* < 0.0001 ALP vs. ALP + DSF and DSF vs. ALP + DSF.

Serum uric acid levels were evaluated in all rats before and after treatment to assess the therapeutic impact on hyperuricemia (Figure 3). Hyperuricemic rats received oral therapy for three weeks. Remarkably, Group V demonstrated a significant reduction in uric acid levels compared to Group III (*p* < 0.05), with an even greater decrease observed when compared to Group IV (*p* < 0.01). Specifically, Group V achieved a striking 71.43% reduction in serum uric acid, far surpassing the reductions seen in Group III (42.86%) and Group IV (57.14%) (*p* < 0.001), highlighting the superior efficacy of the combination therapy (Figure 3).

#### 2.2.2. Effect of ALP with DSF on Pro-Inflammatory Cytokines

**Figure 4:**
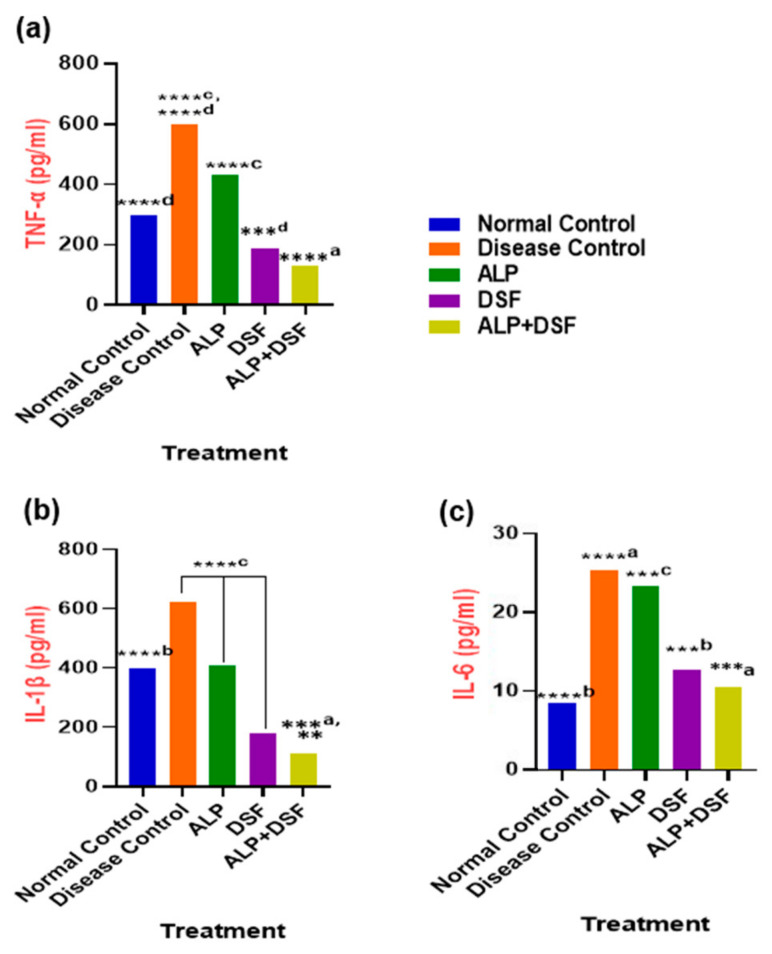
(**a**) TNF-α, (**b**) IL-1β, and (**c**) IL-6 in PO and MSU-induced GAR. Values are expressed as mean ± SEM, *n* = 6. Data were analyzed by One-way ANOVA followed by Tukey’s multiple comparison test. ** *p* < 0.05 DSF vs. ALP + DSF, ***^a^ *p* < 0.001 ALP vs. ALP + DSF, ***^b^ *p* < 0.001 Normal Control vs. DSF, ***^c^ *p* < 0.001 ALP vs. DSF, ***^d^ *p* < 0.001 DSF vs. ALP + DSF, ****^a^ *p* < 0.0001 Disease Control vs. ALP + DSF, ****^b^ *p* < 0.0001 Normal Control vs. ALP, ****^c^ *p* < 0.0001 Disease Control vs. ALP + DSF, ALP vs. DSF and DSF vs. ALP + DSF, ****^d^ *p* < 0.0001 Disease Control vs. ALP and Normal Control vs. ALP + DSF.

Pro-inflammatory cytokines were significantly elevated during acute gouty arthritis. As shown in Figure 4a–c, the levels of key pro-inflammatory cytokines (IL-1β, TNF-α, and IL-6) were assessed. After this study, animals in Group V (treated with ALP and DSF) exhibited a substantial reduction in pro-inflammatory cytokines (*p* < 0.001) compared to both Group III and Group IV. These findings suggest that the drug administration effectively alleviates symptoms of MSU-induced gouty arthritis. Specifically, ALP treatment led to a 28.33% decrease in TNF-α levels, demonstrating a moderate anti-inflammatory effect, and a 33.87% reduction in IL-1β levels, indicating moderate anti-inflammatory activity. However, ALP treatment resulted in only a modest 8.33% reduction in IL-6 levels. On the other hand, DSF treatment showed more pronounced effects, significantly reducing TNF-α levels by 68.33% and IL-1β levels by 70.97%. Additionally, DSF treatment led to a notable 50% reduction in IL-6 levels. Notably, the combination of ALP and DSF exhibited the highest efficacy, reducing TNF-α levels by 78.33%, IL-1β levels by 82.26%, and IL-6 levels by 58.33% compared to the Disease Control group, underscoring its superior anti-inflammatory action.

### 2.3. Assessment of Renal Function

#### Effect of ALP with DSF on Renal Function

At the end of this study, serum urea and creatinine levels were measured. Group V showed a significant reduction in serum urea levels (*p* < 0.001) compared to Group III and Group IV (Table 4). In Group IV, creatinine levels were significantly reduced (*p* < 0.01), indicating a modest improvement in kidney function but not a complete return to normal. In contrast, Group V demonstrated superior effectiveness by significantly (*p* < 0.05) reducing kidney inflammation and lowering the urate burden, leading to near-normal kidney function. The DSF group reduced serum urea by 29.14% compared to the Disease Control group, while the ALP group showed a moderate 15.92% reduction, suggesting partial improvement. However, the combination of ALP and DSF (Group V) led to the most significant progress, with a 32.89% decrease in serum urea. For creatinine, Group II (ALP) showed a 30.83% reduction, Group IV (DSF) had a 46.62% decrease, and Group V (ALP + DSF) achieved the highest reduction of 54.89%, suggesting a synergistic effect of the combined treatment.

### 2.4. Radiographic Studies

**Figure 5:**
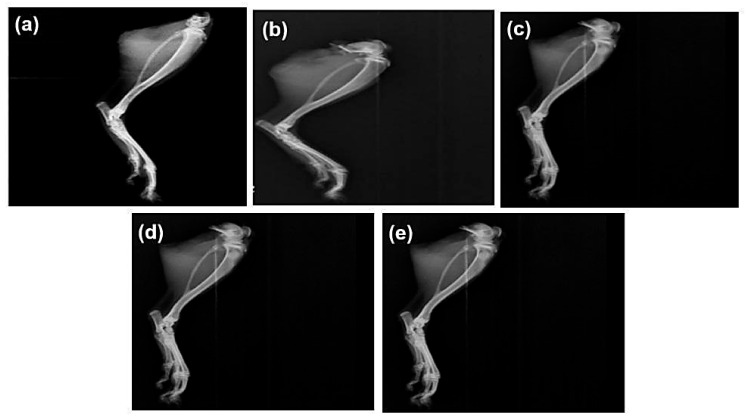
Effect of ALP and DSF on radiographs of the ankle joint in PO and MSU-induced GAR of (**a**) normal control, (**b**) disease control, (**c**) ALP (10 mg/kg), (**d**) DSF (50 mg/kg), and (**e**) ALP (10 mg/kg) + DSF (50 mg/kg).

Radiographic images of the ankle joints of all groups were observed at the end of the treatment period (Figure 5). Radiographic alterations are diagnostic tests that reveal the severity of the condition. Soft tissue swelling at the hind paw indicates moderate erosion of the ankle joint in the disease control group. This swelling and moderate erosion may be due to the accumulation of MSU crystals in the ankle joint. The ALP with DSF-treated group shows no hind paw swelling and mild erosion.

### 2.5. Histopathological Evaluation

**Figure 6:**
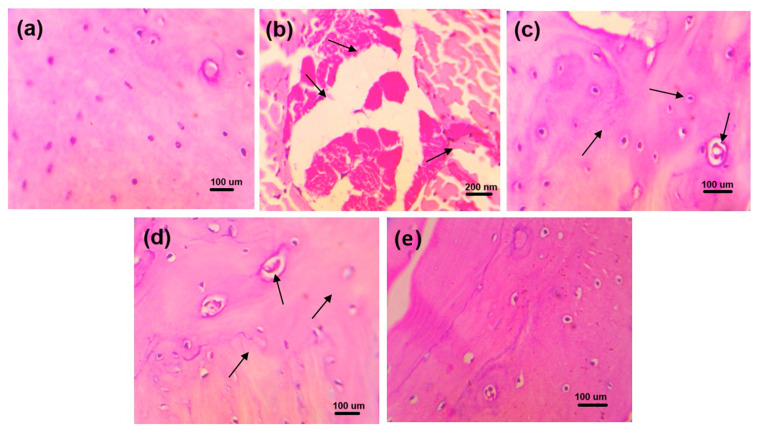
Histopathological changes in ankle joints of experimental animals of (**a**) normal control, (**b**) disease control, (**c**) ALP (10 mg/kg), (**d**) DSF (50 mg/kg), and (**e**) ALP (10 mg/kg) + DSF (50 mg/kg).

The histopathological studies of the ankle joint showed that the periosteum of the ankle joints and cartilage in normal control rats showed normal structure and no sign of deterioration (Figure 6). In disease control experiments, the ankle joints of rats showed clear signs of pathology, chondrocyte degeneration, synovial thickening, and inflammatory cell infiltration. The ALP rats showed (a) mild changes in chondrocyte degeneration, (b) synovial thinning, and (c) inflammatory cell infiltration. DSF-treated rats showed (a) mild changes in chondrocyte degeneration, (b) synovial thinning, and (c) reduction in inflammation. Moreover, the ALP with DSF-treated rats, showed regeneration of chondrocytes and mild changes in synovial lining resembling normal histological structure.

**Figure 7:**
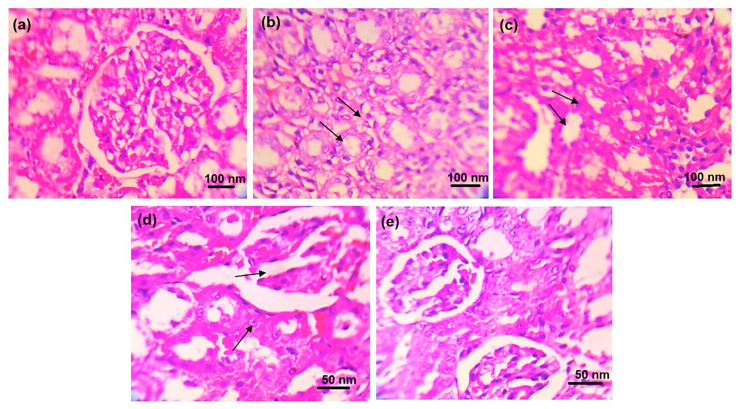
Histopathological changes in the kidney of experimental animals of (**a**) normal control, (**b**) disease control, (**c**) ALP (10 mg/kg), (**d**) DSF (50 mg/kg), and (**e**) ALP (10 mg/kg) + DSF (50 mg/kg).

The rats in the healthy control group displayed normal glomerular and tubular structure, according to the histological test of the kidney (Figure 7). The kidneys of the rats under disease control showed clear pathological alterations, including (a) obstruction of tubular lumina by cell infiltration and (b) atrophy of glomerular tufts with interstitial round cell infiltration and periglomerular. The rats treated with ALP exhibited (a) minor alterations in tubular lumina blockage by dense cell infiltration and (b) minor expansion of glomerular tufts. Rats given DSF exhibited (a) less dense cell infiltration occluding tubular lumina and (b) glomerular tuft expansion. Additionally, rats given ALP and DSF demonstrated repair of the glomerular and tubular anatomy.

### 2.6. Effect of Treatment on NLRP3 Protein Expression

**Figure 8:**
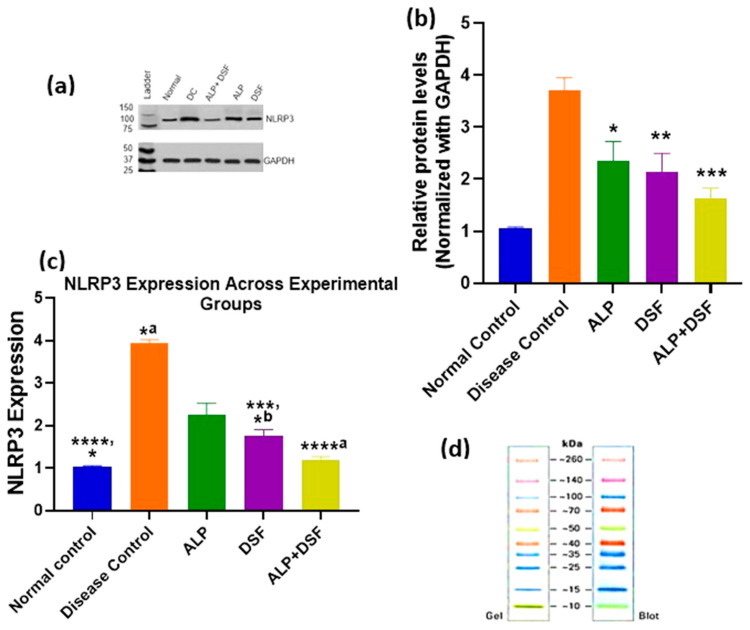
Comparative analysis of NLRP3 expression across treatment groups using Western blot. Values are expressed as mean ± SEM, *n* = 6. Data were analyzed by One-way ANOVA followed by Tukey’s multiple comparison test. (**a**) Western blot analysis of NLRP3 expression in treatment groups. (**b**) Densitometric analysis of NLRP3 levels normalized to GAPDH. (* *p* < 0.05 ALP vs. Disease control, ** *p* < 0.01 DSF vs. Disease control, *** *p* < 0.001 ALP + DSF vs. Disease control.) (**c**) Graphical representation of NLRP3 protein expression levels across experimental groups. **** *p* < 0.0001 Normal control vs. Disease control, ****^a^ *p* < 0.0001 Disease control vs. ALP + DSF, *** *p* < 0.001 Disease control vs. DSF, * *p* < 0.05 Normal Control vs. ALP, *^a^ *p* < 0.05 Disease Control vs. ALP, *^b^ *p* < 0.05 Normal Control vs. DSF. (**d**) Protein ladder reference image (Gel and Blot view).

NLRP3 protein expression was assessed by Western blot and normalized to GAPDH (Figure 8b). Quantitative analysis of band intensities across experimental groups is presented in Figure 8c. A marked upregulation of NLRP3 was observed in the Disease Control group compared to the Normal Control (**** *p* < 0.0001), indicating pronounced inflammasome activation. Treatment with all active agents resulted in a significant reduction in NLRP3 levels. Specifically, ALP monotherapy reduced expression significantly (*^b^ *p* < 0.05 vs. Disease Control), while DSF showed a more substantial decrease (*** *p* < 0.001 vs. Disease Control; *^a^ *p* < 0.05 vs. Normal Control). Notably, the combination of ALP and DSF led to the most profound suppression of NLRP3 expression (****^a^ *p* < 0.0001 vs. Disease Control), restoring levels close to those observed in the Normal Control group. ALP alone also showed a modest but statistically significant difference compared to the Normal Control (* *p* < 0.05), indicating partial restoration. These findings highlight the enhanced anti-inflammatory effect of the combination therapy, suggesting a synergistic suppression of NLRP3 inflammasome activation compared to individual treatments.

## 3. Discussion

Gouty arthritis is a complex inflammatory disorder driven by monosodium urate (MSU) crystal deposition and sustained hyperuricemia. While conventional urate-lowering therapies (ULTs) such as allopurinol (ALP) effectively reduce serum urate levels, they fail to adequately suppress the inflammatory cascade that exacerbates joint damage and triggers recurrent flares [1]. NLRP3 inflammasome activation has emerged as a central mechanism in gout pathogenesis, presenting a therapeutic target beyond urate control [2]. This study evaluated the synergistic therapeutic potential of ALP and disulfiram (DSF), a known NLRP3 inhibitor in a potassium oxonate (PO) and MSU-induced gouty arthritis model in Wistar rats.

In this model, PO served as a uricase inhibitor to induce hyperuricemia, while MSU crystal injection mimicked acute gouty inflammation [25]. The activation of the NLRP3 inflammasome within innate immune cells primarily drives the inflammatory cascade. Upon phagocytosis by macrophages, MSU crystals trigger lysosomal destabilization, potassium efflux, and elevated production of reactive oxygen species (ROS), well-established upstream signals that converge to promote NLRP3 inflammasome assembly. Once activated, NLRP3 recruits the adaptor protein ASC and pro-caspase-1 to form a functional inflammasome complex, which cleaves pro-caspase-1 into its active form. This processes pro-IL-1β into mature IL-1β, a key pro-inflammatory cytokine that initiates a cascade involving neutrophil recruitment, endothelial activation, and the upregulation of additional mediators such as TNF-α and IL-6 [26,27]. Our results demonstrated that while ALP monotherapy significantly lowered serum urate, its effect on inflammatory cytokines was modest. In contrast, DSF—alone or combined with ALP—significantly reduced IL-1β, TNF-α, and IL-6 levels (Figure 4), resulting in better joint preservation and systemic inflammation control. These findings confirm that dual targeting of hyperuricemia (via ALP) and inflammasome-driven inflammation (via DSF) yields superior therapeutic outcomes.

The combination regimen also demonstrated improvements in clinical parameters, including inflammation index, paw swelling, and dysfunction scores (Figure 1 and Figure 2, Table 2), with the ALP + DSF group showing the most significant benefit (*p* < 0.01). This therapeutic synergy aligns with prior study showing that DSF directly inhibits NLRP3 inflammasome activation [28,29], while ALP primarily reduces urate-induced oxidative stress [30]. Further, the disease control group (Group II) exhibited the highest values across all three markers, confirming severe MSU-induced inflammation, consistent with previous findings that MSU crystal deposition leads to NLRP3 inflammasome activation and promotes cytokine release [31]. Allopurinol alone (Group III) showed only partial effectiveness, as evidenced by moderate reductions in inflammation index (Figure 2) and paw swelling (Figure 1). In contrast, dysfunctional index scores (Table 3) remained elevated in Grades 2 and 3, which aligns with studies showing that allopurinol primarily reduces oxidative stress but does not fully suppress pro-inflammatory pathways [27]. In contrast, disulfiram (Group IV) demonstrated a more substantial reduction in all three markers, suggesting its direct role in suppressing NLRP3 inflammasome [29]. Notably, the allopurinol + disulfiram combination (Group V) demonstrated the most significant improvement (*p* < 0.01) across all inflammation parameters, confirming a synergistic therapeutic effect.

These findings were further substantiated by our Western blot analysis, which revealed significant downregulation of NLRP3 protein expression in the DSF and ALP + DSF treatment groups (Figure 8), thereby providing direct confirmation of the proposed inflammasome-inhibitory mechanism. Interestingly, although DSF is not traditionally classified as a urate-lowering agent, our results showed a modest yet significant reduction in serum uric acid levels in the DSF-treated group (Figure 3). This effect may be attributed to its indirect influence on renal urate handling and oxidative stress modulation. Supporting this, Tian et al. (2021) demonstrated that DSF suppressed xanthine oxidase (XO) activity and pyroptosis-related renal inflammation in a similar model [29]. Collectively, these findings suggest a dual role for DSF in modulating both inflammation and urate metabolism, potentially through suppression of the NLRP3/ASC/caspase-1/GSDMD signaling axis.

Hyperuricemia is closely linked to renal dysfunction, as urate crystals can accumulate in the kidneys, causing oxidative stress, inflammation, and nephropathy [30]. In the present study, renal protection was further supported by reduced creatinine and urea levels in the ALP + DSF group (Table 4), indicating mitigation of urate-induced nephropathy. Histological analyses (Figure 6 and Figure 7) confirmed that this combination preserved joint and kidney architecture better than monotherapy, and radiographic evaluation (Figure 5) revealed reduced joint space narrowing and soft tissue swelling. These results are consistent with previous reports on the nephroprotective effects of NLRP3 inhibition in hyperuricemic models [29,32,33].

Interestingly, at the end of this study, animals in Group V (treated with both allopurinol and disulfiram) exhibited a significant increase in final body weight compared to those receiving allopurinol alone (*p* < 0.01). While allopurinol monotherapy was associated with a reduction in body weight relative to baseline, the combination treatment group maintained their initial body weight throughout this study, suggesting that disulfiram may have mitigated treatment-related weight loss. This trend aligns with recent findings by Bernier et al. (2020), who demonstrated that disulfiram prevented and even reversed diet-induced obesity in rodent models [34]. They attributed this effect to enhanced energy expenditure and reduced feeding efficiency without affecting total caloric intake. Although the present model was not designed to evaluate obesity, our findings support the hypothesis that disulfiram contributes to metabolic homeostasis and protects against unintended weight loss, possibly through its anti-inflammatory action and systemic modulation of metabolic signaling.

Disulfiram (DSF), traditionally used to treat alcohol dependence, has recently emerged as a potent inhibitor of the NLRP3 inflammasome, offering a novel immunomodulatory strategy in gout management [35,36,37]. Mechanistically, DSF stabilizes lysosomal membranes and inhibits cathepsin B release, a key upstream activator of NLRP3. It also suppresses intracellular reactive oxygen species (ROS) production, particularly from NADPH oxidase, thereby preventing the assembly and activation of the NLRP3 inflammasome. This cascade results in reduced caspase-1 activation and IL-1β maturation. DSF indirectly attenuates downstream NF-κB signaling and the production of secondary cytokines like TNF-α and IL-6, offering broader anti-inflammatory benefits. These multifaceted actions explain the significant reductions in inflammatory markers, synovial tissue damage, and clinical signs of inflammation observed in DSF-treated animals, supporting its repurposing potential in gout. 

On the other hand, allopurinol exerts its therapeutic effect by inhibiting xanthine oxidase, thereby reducing the formation of uric acid and effectively lowering serum urate levels. However, while this metabolic intervention addresses hyperuricemia, it does not adequately suppress the inflammatory processes initiated by persistent MSU crystal deposition, particularly in patients with recurrent flares [38]. In combination, DSF complements allopurinol’s urate-lowering action by targeting the immunological drivers of gout. This dual approach metabolic regulation by allopurinol and immunomodulation by DSF achieved superior outcomes in cytokine suppression, joint integrity preservation, and renal safety. The combination therapy was well-tolerated, with no observed toxicity, underscoring its potential as a synergistic and clinically viable strategy for comprehensive gout management.

Despite these promising outcomes, several limitations should be acknowledged. This study employed only a single, relatively high dose of DSF (50 mg/kg); future investigations should explore dose–response relationships to establish the optimal therapeutic window and define the safety margins. In addition, pharmacokinetic and drug interaction studies were not conducted; comprehensive profiling encompassing plasma drug concentrations, cytochrome P450 involvement, and transporter-mediated interactions will be essential to ensure this combination’s safety and clinical translatability. While our findings confirmed NLRP3 inflammasome inhibition through Western blot analysis, further molecular validation involving upstream regulators (e.g., ROS, cathepsin B) and downstream components (e.g., caspase-1, GSDMD) could provide more mechanistic depth. Moreover, although DSF demonstrated modest urate-lowering effects, this study did not assess xanthine oxidase activity or the expression of renal and intestinal urate transporters, which are important for understanding its influence on urate homeostasis. Finally, the long-term safety and efficacy of this combination therapy, particularly in individuals with comorbid conditions such as chronic kidney disease or metabolic syndrome, remain to be evaluated in extended preclinical and clinical settings.

These findings highlight the therapeutic advantage of combining allopurinol with disulfiram for more comprehensive management of gouty arthritis. By simultaneously targeting hyperuricemia and the NLRP3-mediated inflammatory cascade, this dual approach attenuates acute inflammatory responses and preserves joint and renal integrity. The synergy between metabolic and immunological modulation suggests that such combinatorial strategies may address current gaps in gout therapy. As disulfiram continues to gain attention for its anti-inflammasome properties, these preclinical results offer a strong rationale for advancing this combination toward clinical evaluation.

## 4. Materials and Methods

### 4.1. Materials

Allopurinol (ALP) was purchased from Harman Finochem Ltd., Mumbai, Maharashtra, India, and DSF was purchased from Laurus Labs, Hyderabad, Telangana, India. Potassium oxonate (PO) was purchased from Tokyo Chemical Industry (TCI) chemicals (Pune, Maharashtra, India). Uric acid and sodium hydroxide were purchased from Loba Chemicals (Mumbai, Maharashtra, India). All the other chemicals used in the current research were of analytical grade.

### 4.2. Synthesis of MSU Crystals

**Figure 9:**
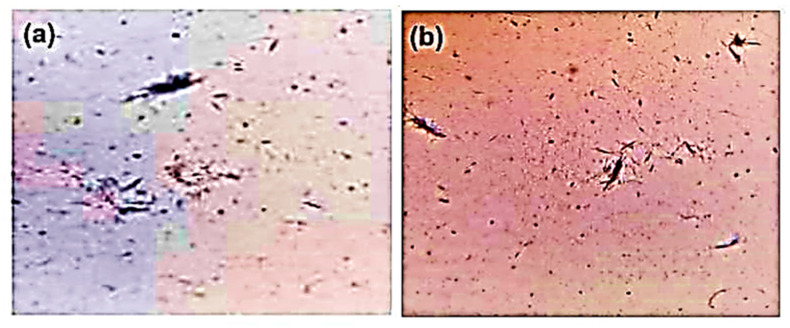
Microscopic image of MSU crystals: (**a**) 20× and (**b**) 10×.

An amount of 4 g of uric acid was dissolved and boiled in 800 mL of H_2_O (dihydrogen monoxide) with NaOH (sodium hydroxide) (9 mL/0.5 N), and adjusted to pH 8.9 at 60 °C. It was then allowed to cool overnight in a cold room before being washed and dried. In sterile saline (25 mg/mL), needle-like crystals (Figure 9) were obtained [5].

### 4.3. Pharmacological Study Design

#### 4.3.1. Selection of Animals

Adult Albino Wister rats in good health (male 150–200 g) were employed. They were obtained from the Central Animal House of Swamy Vivekanandha College of Pharmacy. They were accommodated in polypropylene cages and kept in an air-conditioned, adequately ventilated animal house with accepted lab conditions at temperature (25 ± 2 °C), humidity (50 ± 1.5%), and under a 12 h light/dark cycle. They were given a typical pellet diet and unlimited access to water. The Institutional Animal Ethics Committee (IAEC) at Swamy Vivekanandha College of Pharmacy, Elayampalayam, Namakkal, approved the experimental protocol. According to the Indian National Science Academy (INSA) guidelines for using and handling experimental animals in research, virtually all the animals utilized in the study were treated humanely. The animal experiments were carried out per Committee for Control and Supervision of Experiments on Animals (CCSEA) guidelines with IAEC Reference No: SVCP/IAEC/PG/4/05/2022.

#### 4.3.2. Animal Grouping

Thirty rats weighing 150–200 g were divided randomly into five groups. Each group contained six animals. The groups and treatments were designed as follows according to Table 5.

#### 4.3.3. Training of Animals

The animals underwent conditioning by doing a once-daily training experiment for seven days without administering any drugs. For the investigation, only fully trained animals were used.

#### 4.3.4. Induction of Gouty Arthritis in Rats

To replicate the progression of human disease, hyperuricemia was first induced by intraperitoneal administration of potassium oxonate (250 mg/kg/day for 21 days) [39], suspended in 0.5% carboxymethyl cellulose sodium (CMC-Na) solution. This metabolic disturbance was followed by the injection of MSU crystals to trigger acute gouty arthritis, replicating the clinical sequence from asymptomatic hyperuricemia to inflammatory gout. On their 22nd day, rats were given an injection of MSU crystals in the sub-plantar tissue of the left hind paw, except for normal controls. In five groups of six rats, this study used PO (250 mg/kg) and monosodium urate crystal (0.2 mL) to induce gouty arthritis. Regarding drug treatment, ALP (10 mg/kg) for Group III, DSF (50 mg/kg) for Group IV, and a combination of ALP (10 mg/kg) and DSF (50 mg/kg) for Group V were given. DSF was suspended in 0.5% CMC-Na solution to ensure a uniform and biocompatible oral delivery vehicle. The selection of ALP [40] and DSF [41] doses was based on previously published literature. Following the introduction of gouty arthritis on day 22, the medication therapy began and lasted up to day 30.

### 4.4. Physical Evaluation

#### 4.4.1. Measurement of Bodyweight

Using a weighing balance, the body weight for every rat in each group was first determined on day 0 and day 30, with changes being noted.

#### 4.4.2. Assessment of Paw Swelling

Before and at the end of this study, the paw swelling was used to evaluate the inflammation. Following the injection of MSU crystals on days 21, 26, and 30, the ankle size of each inflamed paw rat was measured with a vernier caliper.

#### 4.4.3. Inflammation Index and Dysfunction Index

To assess the severity of the arthritis, both indices were used. Data were gathered before and following the MSU injection on days 21, 26, and 30. The ratings of rat inflammation and dysfunction were assessed by two independent observers [42].

#### 4.4.4. Methodology to Rate the Inflammation

Grade 0 (0 points): without any indications of inflammation, the ankle joint is healthy. Grade 1 (2 points): the joints have noticeable bony scars, mild edema, and erythematous skin. Grade 2 (4 points): bony markings have vanished, and swelling is confined to the joints, which are red and swollen. Grade 3 (6 points): the foot’s function is hindered, the inflammatory process is more powerful, and the foot frequently lifts off the ground.

#### 4.4.5. Methodology to Score Dysfunction

Grade 0 (0 points): normally, both feet should be evenly on the ground. Grade 1 (2 points): the animal shows a slight limp, and the toes are not fully spread. Grade 2 (4 points): The foot is twisted and has a clear limp. The toes are on the ground. Grade 3 (6 points): a three-legged stride in which the bottom foot is raised [43].

### 4.5. Biochemical Estimation

#### 4.5.1. Estimation of Uric Acid

At the end of the experimental period, blood samples were collected from the retro-orbital plexus of the rats. The samples were allowed to clot at room temperature for 30 min and then centrifuged at 3000 rpm for 15 min to separate the serum. Serum uric acid levels were estimated using a commercially available diagnostic kit based on the phosphotungstic acid method, following the manufacturer’s instructions [39].

#### 4.5.2. Estimation of IL-1β, TNF-α, and IL-6

Following the completion of this study, animals were euthanized humanely by administering an overdose of sodium pentobarbital (150 mg/kg, intraperitoneally), following CCSEA and INSA guidelines for ethical animal use. Serum and joint fluid were collected for cytokine analysis. The concentrations of pro-inflammatory cytokines interleukin-1 beta (IL-1β), tumor necrosis factor-alpha (TNF-α), and interleukin-6 (IL-6) were quantified using commercially available enzyme-linked immunosorbent assay (ELISA) kits from Elabscience (Cat. No: E-EL-H0109 for TNF-α, E-CL-R0012 for IL-1βand E-EL-R0015 for IL-6), following the protocols provided by the respective manufacturers [44].

#### 4.5.3. Assessment of Renal Function

Renal function was evaluated at the end of this study by measuring serum levels of urea and creatinine. Blood samples were collected from the retro-orbital plexus under light anesthesia using sterile capillary tubes. The collected blood was immediately transferred to heparinized tubes and centrifuged at 4000 rpm for 5 min at 4 °C to separate the plasma. Plasma urea levels were determined using the glutamate dehydrogenase (GLDH) kinetic method, while creatinine levels were assessed using the alkaline picrate method. According to the manufacturers’ instructions, both parameters were measured using commercially available biochemical kits [45,46].

#### 4.5.4. Radiographic Analysis

After this study, animals were given ether anesthesia before being placed in an X-ray machine for a radiographic investigation of the tibiotarsal joint. To determine the degree of arthritis in rats with monosodium urate crystal-induced arthritis, an X-ray was performed at the bone joint of the contralateral paw.

### 4.6. Histopathological Evaluation

Rat kidneys were kept in 4% paraformaldehyde at room temperature for 24 h. Each kidney was divided into 4 μm thick paraffin slices, stained with hematoxylin and eosin, and inspected under a 200× light microscope. The MSU crystal-induced gouty arthritis rat paw region was removed, decalcified in a 10% formaldehyde solution, fixed in paraffin, segmented at a thickness of 5 μm, stained with hematoxylin and eosin reagent, and investigated under a light microscope at a magnification of 200×.

### 4.7. Western Blot Analysis

Tissue was collected, and total proteins were extracted using RIPA cell lysis buffer (SKU: AR0105-100, Boster Bio, Pleasanton, CA, USA) and maintained on ice for a minimum of 30 min. Lysates were centrifuged at 12,000× *g* for 10 min at 4 °C, and the supernatant was transferred to a new tube. Protein concentration was determined via the bicinchoninic acid (BCA) assay. Equal amounts of protein were loaded per lane, separated by SDS-PAGE, and transferred to PVDF membranes. Membranes were blocked with 10% bovine serum albumin (BSA) in 0.05% Tris-buffered saline with Tween 20 (TBST) for 1 h at room temperature, followed by overnight incubation at 4 °C with the following antibodies: Anti-NLRP3 antibody [EPR23094-149]—Abcam #ab263899, and Rabbit GAPDH (14C10) Rabbit mAb #2118 (Cell Signaling, R&D Systems, Catalog # PPS072). After three washes, membranes were incubated with HRP-conjugated Goat anti-Rabbit IgG (H + L) (#31460) for 2 h at room temperature. Detection was carried out using enhanced chemiluminescence (ECL) reagents (Pierce, Rockford, IL, USA) and visualized.

### 4.8. Statistical Analysis

The data were represented as mean ± SEM of six replicated determinations. The results were analyzed statistically by One-way analysis of variance (ANOVA) followed by Tukey’s multiple comparisons. The differences were considered significant at *p* < 0.001 (***, a), *p* < 0.01 (**, b), or *p* < 0.05 (*, c). All statistical tests were carried out using Prism 8.0 (Graph Pad, San Diego, CA, USA) statistical software.

## 5. Conclusions

This study provides compelling evidence that combining allopurinol with disulfiram offers a superior therapeutic strategy for gouty arthritis. This combination therapy could reduce flare severity, prevent joint damage, and improve long-term outcomes by addressing both urate metabolism and inflammasome-driven inflammation. Given the growing interest in NLRP3 inhibitors, DSF presents a promising, clinically viable alternative for inflammation control in gout. Future studies should further evaluate its translational potential in human trials, paving the way for novel combination therapies in inflammatory arthritis.

## Figures and Tables

**Table 1:** Effect of ALP with DSF on body weight changes in PO and MSU-induced GAR.

S.No	Treatment	Before Treatment	After Treatment
1	Group I (Normal Control)	175.8 ± 1.721	180.0 ± 0.5774 ****^a,^ ****^b,^
2	Group II (Disease Control)	180.7 ± 1.430	128.5 ± 2.232 ****^c^, ****^d^, ****^e^
3	Group III (ALP)	171.3 ± 4.022	159.7 ± 1.358 **
4	Group IV (DSF)	181.3 ± 2.028	177.3 ± 1.116
5	Group V (ALP + DSF)	180.2 ± 2.358	179.7 ± 1.453 ***

Values are expressed as mean ± SEM, *n* = 6. Data were analyzed using One-way ANOVA followed by Tukey’s multiple comparison test. ****^a^ *p* < 0.0001 Normal Control vs. Disease Control, ****^b^ *p* < 0.0001 Normal Control vs. ALP, ****^c^ *p* < 0.0001 Disease Control vs. ALP, ****^d^ *p* < 0.0001 Disease Control vs. DSF, ****^e^ *p* < 0.0001 Disease Control vs. ALP + DSF, ** *p* < 0.01 Disease Control vs. ALP + DSF, *** *p* < 0.01 ALP vs. ALP + DSF.

**Table 2:** Effect of ALP with DSF on assessment of paw swelling in PO and MSU-induced GAR.

S. No	Treatment	Paw Swelling (Mm)		
		Day 21	Day 26	Day 30
1.	Group I (Normal Control)	3.44 ± 0.07 *^a^	3.44 ± 0.07 ****^a^, ****^b^	3.25 ± 0.10 ****^a^, ****^b^
2.	Group II (Disease Control)	3.44 ± 0.09 *^b^	6.78 ± 0.65 *^c,^ **	6.35 ± 0.62 ***^c^
3.	Group III (ALP)	3.54 ± 0.06	5.96 ± 0.18 ***^b^, ****^c^	4.70 ± 0.06 ****^c^
4.	Group IV (DSF)	3.55 ± 0.18	5.53 ± 0.14 ****^d^	4.15 ± 0.13 ****^d^
5.	Group V (ALP + DSF)	3.52 ± 0.08	4.72 ± 0.05 ***^a^	3.37 ± 0.02 *^d^

Values are expressed as mean ± SEM, *n* = 6. Data were analyzed by One-way ANOVA followed by Tukey’s multiple comparison test. *^a^ *p* < 0.05 Normal Control vs. ALP, *^b^ *p* < 0.05 Disease Control vs. ALP, *^c^ *p* < 0.05 Disease Control vs. ALP, *^d^ *p* <0.05 Normal Control vs. ALP + DSF, ****^b^ *p* < 0.0001 Normal Control vs. ALP, ** *p* < 0.05 Disease Control vs. DSF, ***^a^ *p* < 0.001 Disease Control vs. ALP + DSF, ***^b^ *p* < 0.001 ALP vs. DSF, ***^c^ *p* < 0.001 Disease Control vs. ALP, ****^a^ *p* < 0.0001 Normal Control vs. Disease Control, ****^c^ *p* < 0.0001 ALP vs. ALP + DSF, ****^d^ *p* < 0.0001 DSF vs. ALP + DSF.

**Table 3:** Effect of ALP with DSF on the dysfunction index in PO and MSU-induced GAR.

S.NO	Treatment	Unit	After Injection of MSU Crystal							
			On Day 26				On Day 30			
			Grade 0	Grade 1	Grade 2	Grade 3	Grade 0	Grade 1	Grade 2	Grade 3
1.	Group I (Normal Control)	pcs	0.00 ± 0.00	0.00 ± 0.00 **^a^	0.00 ± 0.00	0.00 ± 0.00	0.00 ± 0.00	0.00 ± 0.00 **^a^	0.00 ± 0.00	0.00 ± 0.00
2.	Group II (Disease Control)	pcs	0.00 ± 0.00	1.33 ± 0.33 *^a^	3.66 ± 0.21 ****^a^	5.66 ± 0.21 ***^a^	0.00 ± 0.00	1.16 ± 0.30 **^e^	2.83 ± 0.16 ****^a^	4.33 ± 0.33 ****^a^
3.	Group III (ALP)	pcs	0.00 ± 0.00	0.66 ± 0.21 *^b^	2.83 ± 0.16	4.50 ± 0.22 ***^b^	0.00 ± 0.00	0.86 ± 0.21	2.33 ± 0.42 ***^b^	3.25 ± 0.25 ****^b^
4.	Group IV (DSF)	pcs	0.00 ± 0.00	1.16 ± 0.30	2.50 ± 0.22 **^b^	4.16 ± 0.16 **^b^	0.00 ± 0.00	0.83 ± 0.21 **^d^	1.50 ± 0.36 **^b^	2. ± 0.33 ****^c^
5.	Group V (ALP + DSF)	pcs	0.00 ± 0.00	1.00 ± 0.25 *^c^	2.66 ± 0.21 **^c^	2.25 ± 0.47 ***^d^	0.00 ± 0.00	0.50 ± 0.22 ****^b^	0.10 ± 0.10 ***^c^	1.27 ± 0.33 ***^c^

Values are expressed as mean ± SEM, *n* = 6. Data were analyzed by One-way ANOVA followed by Tukey’s multiple comparison test. *^a^ *p* < 0.05—Disease Control vs. ALP + DSF, *^b^ *p* < 0.05 ALP vs. DSF, *^c^ *p* < 0.05 ALP vs. ALP + DSF, **^a^ *p* < 0.05 Normal Control vs. Disease Control, **^b^ *p* < 0.05 ALP vs. DSF, **^c^ *p* < 0.05 ALP vs. ALP + DSF, **^d^ *p* < 0.05 DSF vs. ALP + DSF, **^e^ *p* < 0.05 Disease Control vs. ALP + DSF, ***^a^ *p* < 0.001 Disease Control vs. ALP + DSF, ***^b^ *p* < 0.001 ALP vs. ALP + DSF, ***^c^ *p* < 0.001 DSF vs. ALP + DSF, ***^d^ *p* < 0.001 DSF vs. ALP + DSF, ****^a^ *p* < 0.0001 Disease Control vs. ALP + DSF, ****^b^ *p* < 0.0001 ALP vs. ALP + DSF, ****^c^ *p* < 0.0001 ALP vs. DSF.

**Table 4:** Effect of ALP with DSF on renal function in PO and MSU-induced GAR.

S. No	Treatment	Urea (mg/dL)	Creatinine (mg/dL)
1.	Group I (Normal Control)	45.60 ± 1.03 ****^a^, ****^b^, **^a^	0.50 ± 0.03 ****^a^, **^a^, **^b^, ***^a^
2.	Group II (Disease Control)	69.82 ± 0.61 ****^c^	1.33 ± 0.17 *^a^, ***^b^, ***^c^
3.	Group III (ALP)	58.70 ± 0.25	0.92 ± 0.19 **^c^
4.	Group IV (DSF)	49.47 ± 0.39 ****^d^	0.71 ± 0.14
5.	Group V (ALP + DSF)	46.85 ± 0.23 ****^e^	0.60 ± 0.04 *^b^

Values are expressed as mean ± SEM, *n* = 6. Data were analyzed by One-way ANOVA followed by Tukey’s multiple comparison test. ****^a^ *p* < 0.0001 Normal Control vs. Disease Control, ****^b^ *p* < 0.0001 Normal Control vs. ALP, ****^c^ *p* < 0.0001 Disease Control vs. ALP, ****^d^ *p* < 0.0001 ALP vs. DSF, ****^e^ *p* < 0.0001 DSF vs. ALP + DSF, ***^a^ *p* < 0.001 Normal Control vs. ALP + DSF, ***^b^ *p* < 0.001 Disease Control vs. DSF, ***^c^ *p* < 0.001 Disease Control vs. ALP + DSF, **^a^ *p* < 0.01 Normal Control vs. ALP, **^b^ *p* < 0.01 Normal Control vs. DSF, **^c^ *p* < 0.01 ALP vs. ALP + DSF, *^a^ *p* < 0.05 Disease Control vs. ALP, *^b^ *p* < 0.05 DSF vs. ALP + DSF.

**Table 5:** Animal grouping with the treatment regimen.

S.No	Groups		Treatment	Animals Required
1	Group I	Normal control	Saline 0.5 mL p.o	6
2	Group II	Disease control	MSU crystals 0.2 mL (25 mg/mL) sub-plantar + PO 250 mg/kg i.p	6
3	Group III	ALP Treatment	MSU crystals 0.2 mL (25 mg/mL) sub-plantar +PO 250 mg/kg i.p + ALP 10 mg/kg p.o	6
4	Group IV	DSF treatment	MSU crystals 0.2 mL (25 mg/mL) sub-plantar + PO 250 mg/kg i.p + DSF 50 mg/kg p.o	6
5	Group V	ALP + DSF	MSU crystals 0.2 mL (25 mg/mL) sub-plantar + PO 250 mg/kg i.p + ALP 10 mg/kg p.o + DSF 50 mg/kg p.o	6
		Total number of animals		**30**

## Data Availability

The original contributions presented in this study are included in the article. Further inquiries can be directed to the corresponding author.
